# The Mitochondrial Genomes of the Nudibranch Mollusks, *Melibe leonina* and *Tritonia diomedea*, and Their Impact on Gastropod Phylogeny

**DOI:** 10.1371/journal.pone.0127519

**Published:** 2015-05-21

**Authors:** Joseph L. Sevigny, Lauren E. Kirouac, William Kelley Thomas, Jordan S. Ramsdell, Kayla E. Lawlor, Osman Sharifi, Simarvir Grewal, Christopher Baysdorfer, Kenneth Curr, Amanda A. Naimie, Kazufusa Okamoto, James A. Murray, James M. Newcomb

**Affiliations:** 1 Department of Biology and Health Science, New England College, Henniker, New Hampshire, United States of America; 2 Department of Biological Sciences, University of New Hampshire, Durham, New Hampshire, United States of America; 3 Department of Biological Sciences, California State University, East Bay, Hayward, California, United States of America; Sichuan University, CHINA

## Abstract

The phylogenetic relationships among certain groups of gastropods have remained unresolved in recent studies, especially in the diverse subclass Opisthobranchia, where nudibranchs have been poorly represented. Here we present the complete mitochondrial genomes of *Melibe leonina* and *Tritonia diomedea* (more recently named *T*. *tetraquetra*), two nudibranchs from the unrepresented Cladobranchia group, and report on the resulting phylogenetic analyses. Both genomes coded for the typical thirteen protein-coding genes, twenty-two transfer RNAs, and two ribosomal RNAs seen in other species. The twelve-nucleotide deletion previously reported for the cytochrome oxidase 1 gene in several other *Melibe* species was further clarified as three separate deletion events. These deletions were not present in any opisthobranchs examined in our study, including the newly sequenced *M*. *leonina* or *T*. *diomedea*, suggesting that these previously reported deletions may represent more recently divergent taxa. Analysis of the secondary structures for all twenty-two tRNAs of both *M*. *leonina* and *T*. *diomedea* indicated truncated d arms for the two serine tRNAs, as seen in some other heterobranchs. In addition, the serine 1 tRNA in *T*. *diomedea* contained an anticodon not yet reported in any other gastropod. For phylogenetic analysis, we used the thirteen protein-coding genes from the mitochondrial genomes of *M*. *leonina*, *T*. *diomedea*, and seventy-one other gastropods. Phylogenetic analyses were performed for both the class Gastropoda and the subclass Opisthobranchia. Both Bayesian and maximum likelihood analyses resulted in similar tree topologies. In the Opisthobranchia, the five orders represented in our study were monophyletic (Anaspidea, Cephalaspidea, Notaspidea, Nudibranchia, Sacoglossa). In Gastropoda, two of the three traditional subclasses, Opisthobranchia and Pulmonata, were not monophyletic. In contrast, four of the more recently named gastropod clades (Vetigastropoda, Neritimorpha, Caenogastropoda, and Heterobranchia) were all monophyletic, and thus appear to be better classifications for this diverse group.

## Introduction

Gastropod mollusks are the second most diverse class of metazoans including over 60,000 identified species divided into over 600 families [1]. Additionally, the class is among the few to inhabit terrestrial, freshwater, and marine habitats [2]. Traditionally, the class has been divided into three subclasses, Opisthobranchia, Pulmonata, and Prosobranchia [1, 3]. The legitimacy of this classification has been questioned and scientists have alternatively begun to distinguish five groups within Gastropoda: Patellogastropoda, Vetigastropoda, Neritimorpha, Caenogastropoda, and Heterobranchia [4–8]. Heterobranchia is further divided into the opisthobranchs, pulmonates, and lower Heterobranchia, with some taxonomists grouping the pulmonates and opisthobranchs together in a clade known as Euthyneurans [6, 7, 9–11].

Of all gastropod groups, Opisthobranchia is the most morphologically and ecologically diverse. There are an estimated 5,000 species which are divided into nine orders: Acochlidea, Anaspidea, Cephalaspidea, Gymonostomata, Notaspidea, Nudibranchia, Rhodopemorpha, Sacoglossa, and Thecostomata [12, 13], although these classifications and the phylogenetic relationships between groups still remain uncertain. Nudibranchia is the most speciose of these opisthobranch clades and it has been further divided into two groups, Cladobranchia and Anthobranchia [1, 13]. Nudibranchs have not been well represented in recent molecular studies.

Gastropod mollusks have been previously classified based on a wide range of criteria including morphological characters [2, 14, 15], mitochondrial and nuclear genes [4, 16, 17], mitochondrial gene order [18, 19], and complete mitochondrial genomes [6, 8, 20]. Mitochondrial genes, in particular the thirteen protein-coding genes, have been shown to be especially useful in constructing phylogenetic inferences in extremely diverse clades such as arthropods [21] and Cypriniformes [22]. Although some recent studies using mitochondrial genes to investigate molluscan phylogeny have been problematic [23, 24], it has been demonstrated that an increased sample size and appropriate phylogenetic models can increase the confidence in deep evolutionary relationships [8]. Additionally, the use of complete mitochondrial genomes (typically between 10,000 and 20,000 bp) to investigate phylogenetic inferences offers greater confidence than much shorter single genes [25, 26]. Recent innovations have significantly accelerated the process and reduced the cost of sequencing, and thus many gastropod mitochondrial genomes have recently been published and are now available to increase the size of phylogenetic datasets [5, 6, 8, 20, 26–29].

Here we present the complete mitochondrial genomes of *Melibe leonina* and *Tritonia diomedea* (more recently named *T*. *tetraquetra* [30]), two nudibranchs from the previously under-represented Cladobranchia group. Partial mitochondrial cytochrome oxidase 1 (COX1) sequences for four other species of *Melibe* have been previously reported [13]. These sequences have several nucleotide deletions that are not present in any other examined gastropod [13]. Unlike base substitutions, deletions would potentially alter the codon frame and resulting protein sequence of COX1, making these deletions in the *Melibe* genus highly surprising. As a result, we determined to pay close attention to COX1 in both of our nudibranch species, especially *M*. *leonina*. Furthermore, phylogenetic analyses using the protein-coding genes of mitochondrial genomes were completed with opisthobranch and gastropod data sets to investigate evolutionary relationships and taxonomic groupings.

## Materials and Methods

### Animal collection and housing

Specimens of *M*. *leonina* were collected near Monterey, CA, from the area of Del Monte Kelp Beds, N 36°60’, W 121°88’, by Monterey Abalone Company. All collections of *M*. *leonina* were made outside of any Marine Protected Areas and no specific permission was required to collect *M*. *leonina*. *M*. *leonina* is not on the Prohibited Species list in the California Commercial Fishing Digest of Laws and Regulations 2014/15. Monterey Abalone Company has a Marine Aquaria Collectors Permit, Commercial Fishing License, and Commercial Fish Business License issued by the California Department of Fish and Wildlife. Collected animals were shipped to the University of New Hampshire (UNH). Animals were housed in recirculating tanks containing seawater obtained from the UNH Coastal Marine Lab in New Castle, NH. The seawater was maintained at 10°C and the daily lighting regime consisted of twelve hours of light with a compact fluorescent bulb, followed by twelve hours of darkness.

Specimens of *T*. *diomedea* were collected at Yellow Bank near Tofino, British Columbia, N 49°14.013’, W 125°55.569’, by Living Elements (Vancouver, Canada) in accordance with a permit from Fisheries and Ocean Canada, and shipped overnight in seawater where they were maintained in recirculating tanks at California State University, East Bay (Hayward, CA). After neural recordings from the brain in the semi-intact animal, the brain was removed for processing, and the buccal mass was removed and placed in a zip-lock bag, and placed in a—80°C freezer.

### DNA isolation, sequencing, and assembly

#### Melibe leonine

DNA isolation and sequencing were done twice, on two separate individuals—once in 2011 and again in 2012. In each case, DNA was isolated from the entire body tissues of a single *M*. *leonina* using a Qiagen Genomic Tip 20/G kit. DNA sequencing was done on an Illumina HiSeq1000 platform from shotgun genomic libraries generated using the TrueSeq protocol (Illumina). The assembly was based on 601,323,696 paired-end reads 76 bp in length. The libraries had an estimated insert size of 50 to 500 bp. Paired-end raw reads from both rounds of sequencing were uploaded to CLC Genomics Workbench v7 and assembled together using the following parameters: minimum contig length of 500 bp, mismatch cost of 2, insertion cost of 3, deletion cost of 3, length fraction of 0.5, and similarity fraction of 0.8. The mitochondrial genome was located within the contiguous sequences of *M*. *leonina* by BLASTing the contig assembly with the *M*. *leonina* 16s mitochondrial gene available on GenBank (GU339202.1). BLASTs against closely related species with published mitochondrial genomes on GenBank were used to confirm the identity of the sequence.

#### Tritonia diomedea

The buccal mass was dissected from a slug with scissors and was frozen at—80°C. Total DNA was extracted from tissue with DNeasy Blood and Tissue kit (Qiagen) and suspended in 100ml of AE buffer. Sonicated DNA was used to construct a DNA library using an Ion Plus Fragment Library Kit (Life Technologies). Templated spheres were generated from the library using a OneTouch 200 Template Kit (Life Technologies) and then loaded on two Ion 314 Chips and one Ion 318 Chip (Life Technologies) producing a total of 4.0 million usable sequences with a mean read length of 197 bp. Raw reads were uploaded to CLC Genomics Workbench v6.0.4 and assembled together using the following parameters: minimum contig length of 200 bp, mismatch cost of 2, insertion cost of 3, deletion cost of 3, length fraction of 0.5, and similarity fraction of 0.8. Putative mitochondrial contigs for *T*. *diomedea* were located by BLASTing the contig database with mitochondrial genomes from other opisthobranchs. The complete mitochondrial genome of *T*. *diomedea* was combined by creating a *de novo* assembly using the reads from identified mitochondrial contigs. The final sequence was confirmed by dideoxy sequencing of overlapping PCR-generated mitochondrial amplicons using an Applied Biosystems 3130 Genetic Analyzer.

### Mitochondrial genome annotations

Mitochondrial genome annotations were completed using CLC Genomics Workbench v. 7 open reading frame tool with a specific invertebrate mitochondrial genetic code reader. BLAST searches of open reading frames against NCBI were used to confirm gene identity. Alignments with all available mitochondrial genomes of opisthobranchs were completed using Clustal Omega v 1.2.0 with default settings [31]. Alignments were used to further confirm the location of genes and to identify appropriate start/stop codons. Transfer RNA (tRNA) sequences and secondary structures were identified using Arwen v1.2 [32]. Complete mitochondrial genome fasta files for *M*. *leonina* and *T*. *diomedea* were independently uploaded to ARWEN using the default settings and the invertebrate mitochondrial genetic code. Evidence indicated that the mitochondrial genome sequences had a circular topology and both strands of the genome were searched. BLASTs against NCBI, and previously described alignments with other opisthobranchs, were used to confirm sequences.

### Comparison with publicly available partial sequences for *Melibe* species

Prior to this study, *M*. *leonina* was represented in GenBank with a partial COX1 gene (GQ292059.1) and partial 16s RNA (GU339202.1). The mitochondrial genome sequenced in this study was compared to these data via BLASTs against NCBI and with alignments.

The COX1 genes in four other *Melibe* species (*M*. *arianeae*, *M*. *digitata*, *M*. *rosea*, and *M*. *viridis*) have been previously reported to contain a unique twelve base pair deletion not seen in other nudibranchs [13]. Therefore, the partial COX1 gene from these four species (KC992314, JX306069, JX306074, and JX306075), along with all other available nudibranch COX1 genes (*Chromodoris magnifica—*EU982736, *M*. *leonina—*GQ292059.1, *Notodoris gardineri—*HM162695, *Roboastra europaea—*AY083457, and *T*. *diomedea—*GQ292050), were uploaded from GenBank and aligned to the COX1 gene of *M*. *leonina* from this study using default settings in Clustal Omega v 1.2.0. Since partial COX1 genes were available for the four *Melibe* species on GenBank and complete COX1 genes were available for the remaining species, the uninformative overhang on either side of the complete sequences were trimmed using CLC Genomics Workbench. Raw read coverage support was also examined for the *M*. *leonina* COX1 gene to confirm high coverage underlying any putative deletion sites.

### Alignments and GBlock ambiguity

Complete mitochondrial genomes for all gastropods available on GenBank were downloaded, as well as that of the bivalve, *Venustaconcha ellipsiformis* (FJ809753), which served as an outgroup in the phylogenetic analyses (Table 1). All thirteen protein-coding genes were extracted from the complete mitochondrial genome of each species (based on annotations from GenBank), and translated using CLC Genomics Workbench. The resulting amino acid sequences were combined into a single concatenated sequence using the same gene order ATP8, ATP6, ND3, ND4, COX3, ND2, COX1, ND6, ND5, ND1, ND4L, CYTB, COX2. Additionally, the concatenated nucleotide sequences underlying these protein sequences were used in the analyses of opisthobranchs because they have been shown to increase the confidence in more derived nodes.

**Table 1:** Gastropod mitochondrial genomes used in this study.

Clades		Species	Length	Accession #
**Heterobranchia**				
Opisthobranchia*	Nudibranchia	*Chromodoris magnifica*	14446	DQ991931
		*Melibe leonina*	14513	This study
		*Notodoris gardineri*	14424	DQ991934
		*Roboastra europaea*	14472	AY083457
		*Tritonia diomedea*	14540	This study
	Anaspidea	*Aplysia californica*	14117	AY569552
		*Aplysia dactylomela*	14128	DQ991927
	Cephalaspidea	*Hydatina physis*	14153	DQ991932
		*Micromelo undata*	14160	DQ991933
		*Pupa strigosa*	14189	AB028237
	Notaspidea	*Berthellina ilisima*	15688	DQ991929
	Sacoglossa	*Ascobulla fragilis*	14745	AY345022
		*Elysia chlorotica*	14132	EU599581
		*Placida sp*.	14751	KC171014
Lower Heterobranchia	Pyramidelloidea	*Pyramidella dolabrata*	13856	AY345054
Pulmonata*	Amphibolidea	*Salinator rhamphidia*	14007	JN620539
	Ellobiidea*	*Auriculinella bidentata*	14135	JN606066
		*Myosotella myosotis*	14215	JN606067
		*Ovatella vulcani*	14274	JN615139
		*Pedipes pedipes*	16708	JN615140
	Hygrophila	*Biomphalaria glabrata*	13670	AY380531
		*Biomphalaria tenagophila*	13722	EF433576
		*Galba pervia*	13768	JN564796
	Siphonarioidea	*Siphonaria gigas*	14518	JN627205
		*Siphonaria pectinata*	14065	AY345049
	Stylommatophora	*Albinaria caerulea*	14130	X83390
		*Cepaea nemoralis*	14100	U23045
		*Cornu aspersum*	14050	JQ417194-96
		*Cylindrus obtusus*	14610	JN107636
		*Euhadara herklotsi*	12804	Z71693-701
		*Succinea_putris*	14092	JN627206
	Systellommatophora*	*Onchidella borealis*	14510	DQ991936
		*Onchidella celtica*	14150	AY345048
		*Peronia peronii*	13968	JN619346
		*Platevindex mortoni*	13991	GU475132
		*Rhopalocaulis grandidieri*	14523	JN619347
	Trimusculoidea	*Trimusculus reticulates*	14044	JN632509
**Caenogastropoda**				
Cerithioidea		*Semisulcospira libertina*	15432	KF736848
Littorinimorpha*	Rissooidea	*Oncomelania hupensis*	15182	FJ997214
		*Oncomelania hupensis hupensis*	15186	EU871630
		*Oncomelania hupensis robertsoni*	15191	EU0709378
		*Potamopyrgus antipodarum*	15110	GQ996430
		*Tricula hortensis*	15179	FJ997214
	Tonnoidea	*Cymatium parthenopeum*	15270	EU827200
	Vermetoidea	*Dendropoma gregarium*	15641	HM174252
		*Dendropoma maximum*	15578	HM174253
		*Eualetes tulipa*	15078	HM174254
		*Thylacodes squamigerus*	15544	HM174255
Neogastropoda*	Buccinoidea	*Ilyanassa obsoleta*	15263	DQ238598
		*Nassarius reticulatus*	15271	EU827201
	Cancellarioidea	*Cancellaria cancellata*	16648	EU827195
	Conoidea*	*Conus borgesi*	15536	EU827198
		*Conus consors*	16112	KF887950
		*Conus textile*	15562	DQ862058
		*Fusiturris similis*	15595	EU827197
		*Lophiotoma cerithiformis*	15380	EU440735
		*Terebra dimidiate*	16513	EU827196
	Muricoidea	*Bolinus brandaris*	15380	EU827194
		*Concholepas concholepas*	15495	JQ446041
		*Rapana venosa*	15272	EU170053
		*Reishia clavigera*	15285	NC_010090
		*Thais clavigera*	15285	DQ159954
	Volutoidea	*Amalda northlandica*	15354	GU196685
		*Cymbium olla*	15375	EU827199
**Neritimorpha**				
	Neritoidea	*Nerita melanotragus*	15261	GU810158
**Vetigastropoda**				
	Trochoidea	*Lunella aff*. *Cinerea*	17670	KF700096
		*Tegula brunnea*	17690	JN790613
	Haliotoidea	*Haliotis rubra*	16907	AY588938
		*Haliotis tuberculata tuberculata*	16521	FJ599667
	Fissurelloidea	*Fissurella volcano*	17575	JN790612

(*) indicates a classification that was not supported by this study.

Two separate alignments were built: a nucleotide alignment of the subclass Opisthobranchia and an amino acid alignment for the overall class Gastropoda. Alignments were completed using default settings in Clustal Omega v 1.2.0. For the Opisthobranch nucleotide alignment, a nucleotide substitution saturation analysis was completed using the program DAMBE v5.5 [33]. Prior to the substitution saturation analysis, all incomplete stop codons were removed from the concatenated sequences; this was done to ensure no frame shifts occurred when analyzing the first, second, and third codon positions. The opisthobranch alignment was uploaded to DAMBE and reported as a protein-coding nucleotide sequence with the invertebrate mitochondrial DNA genetic code and all unresolved bases coded as is. Codon positions 1 and 2 were analyzed together and codon position 3 was analyzed separately. For both analyses the proportion of invariant sites were estimated using the Poisson and Invariant distribution, goodness of fit test, and a new tree was created using default settings. After the proportion of invariant sites was established, a measure of substitution saturation analysis was completed. Proportion of invariant sites was entered and all sites were analyzed. This test determined if the observed index of substitution saturation (I_ss_) was significantly lower than the critical I_ss_ (I_ss._c). If so, then the data set is determined to have little saturation and is appropriate for phylogenetic reconstruction. GBlocks v 0.91b was used to identify conserved regions and to remove ambiguity. All settings were left to default except “allowed gap positons” was set to half, that is, sites were removed if more than 50% of the species in the alignment exhibited gaps, missing data, and/or ambiguous bases at any locus in the alignment.

### Phylogenetic analyses

MEGA6 [34] was used to find the best fit model of nucleotide evolution for each alignment by inferring a neighbor-joining tree, using all sites in the alignment, including the first, second, and third codon sites, non-coding positions, and using a very strong branch swap filter. The best fit model for investigating the opisthobranch alignment was the General Time Reversal Model with Gamma distributed and Invariant rates among sites (GTR+G+I) (BIC = 230853.75, #Param = 37, AICc = 230486.02, and lnL = -115206.00). The GTR+G+I model was available in both MEGA6, which we used for maximum likelihood analyses, and Mr. Bayes v3.2.2, which we used for the Bayesian analyses. For the maximum likelihood analyses, the bootstrap method was used to test the resulting phylogeny with a total of 1000 bootstrap replicates. In addition, the gamma rates among sites was set to five discrete gamma categories, all sites were used in the analyses (first, second, and third codon positions, as well as non-coding positions), and the tree inference options were set to nearest neighbor interchange with an automatically generated (NJ/BioNJ) initial tree. The tree with the highest log likelihood (-117790.6095) was considered the best for phylogenetic analysis. For the Bayesian analyses, the following parameters were used: the general form of the nucleotide substitution model was set to 4-by-4 (the standard model with four states—A, C, T(U), and G), the number of substitution types was set to “6”, the GTR model was used, the rates were set to invariable gamma distribution, and all coding sites were sampled. Two runs with four separate MCMC chains were done. Each chain ran for 2,000,000 generations, with the burn-in fraction set to 0.25. All other settings were set to default. When the average standard deviation for split frequencies dropped below 0.01, the two runs were considered well converged. This occurred early in the analyses (around the 20,000^th^ generation), but the analyses were allowed to continue until 2,000,000 total generations were complete. In addition, the potential scale reduction factors (PSRF) and estimated sample size (ESS) indicated convergence. As the two runs approached 2,000,000 generations, the PSRF value was 1.000 (± 0.001) for all tested parameters (branches and nodes, tree length, the six reversible substitution rates, the four stationary state frequencies, the shape of the gamma distribution of rate variation across sites, and the proportion of invariable sites), indicating sufficient convergence of the two runs. The average ESS ranged from 924.24 to 2055.89 (n = 14) for all tested parameters, indicating appropriate sampling. Two files with a total of 8002 trees were generated from the two runs, of which 6002 were sampled (burn-in = 0.25). The tree with the highest probability was considered the most accurate phylogenetic reconstruction. Node support was indicated by posterior probabilities, and branch lengths were measured by substitutions per sight.

For the overall gastropod analysis, the best fit amino acid model available on MEGA6 and Mr. Bayes was the general reversible mitochondrial model with gamma distribution and invariants among sites (mtRev24+G+I; BIC = 198939.147, #Param = 164, AICc = 197289.607, and lnL = –98480.647). Maximum likelihood analyses on MEGA6 were completed with 1000 bootstrap replicates. The amino acid substitution model was set to mtRev+G+I and the gamma rates among sites were set to five discrete gamma categories. No sites were removed from the analyses and the tree inference options were set to nearest neighbor interchange with an automatically-generated initial tree (NJ/BioNJ). The tree with the highest log likelihood (–98512.3917) was considered the best phylogenetic reconstruction. Bayesian Inference on Mr. Bayes was completed using two separate runs for 2,000,000 generations, with the burn-in fraction set to 0.25. The amino acid model was set to mtRev+G+I with fixed frequencies and substitution rates. All other settings were set to default. Similar to above, the runs were considered converged when the standard deviation of split frequencies reached 0.01. In addition, the PSRF value and ESS indicated convergence of the two runs. At 2,000,000 generations, the PSRF value was 1.000 (± 0.04) and the ESS ranged from 900.619 to 1119.058 (n = 74) for all tested parameters, indicating sufficient convergence and appropriate sampling. Two files with a total of 10,802 trees were generated from the two runs, of which, 8102 were sampled. The tree with the highest probability was considered the most accurate phylogenetic reconstruction. Node support was indicated by probabilities, and branch lengths were measured in substitutions per sight.

## Results

### Genomic sequencing and mitochondrial genome structural features

**Fig 1:**
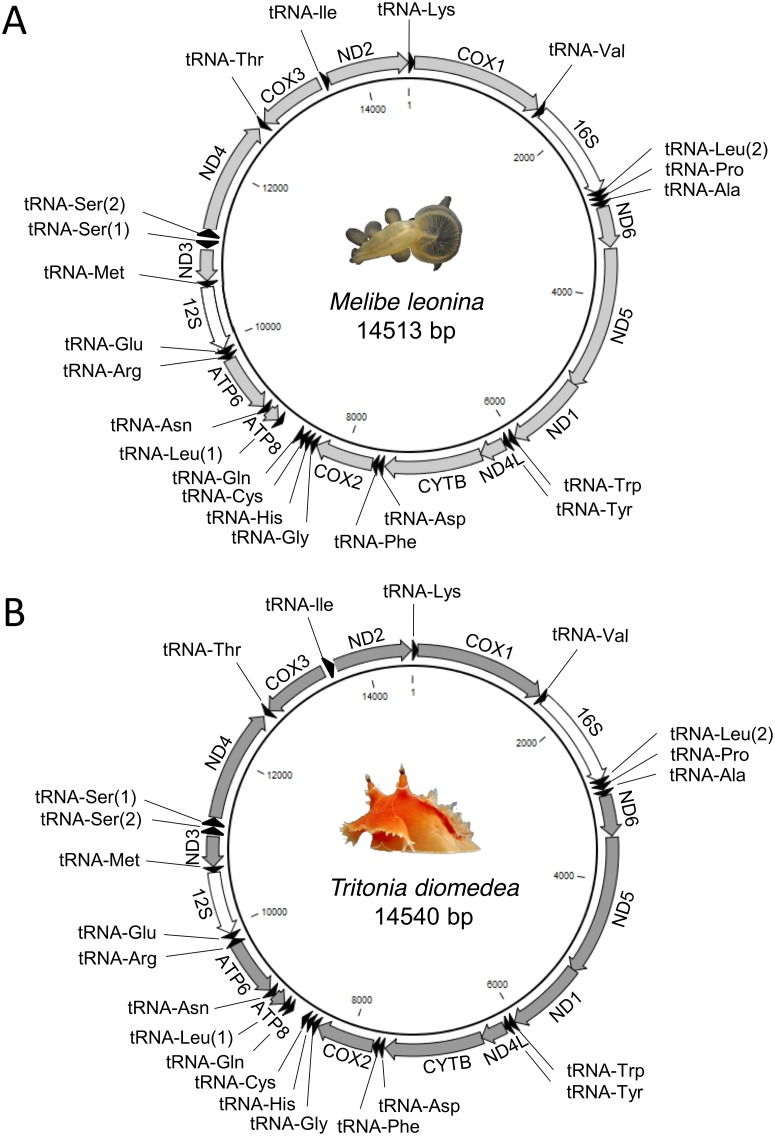
The complete mitochondrial genomes of *Melibe leonina* (A) and *Tritonia diomedea* (B). Both mitochondrial genomes were found to code for the expected 22 transfer RNA, 13 protein-coding genes, and a short and large ribosomal subunit. The 13 protein-coding gene order was found to be identical to all other opisthobranchs.

The resulting *M*. *leonina de novo* assembly contained 225,098 contiguous sequences with an N50 of 1384 base pairs (bp) and greater than 100X average coverage. The overall nuclear genome size was 272 Mbp. The complete mitochondrial genome of *M*. *leonina* was located on a single contiguous sequence that was 14,513 bp in length (Fig 1A; KP764764). The average coverage of the contiguous sequence was 3036X with a total read count of 610,149. A region located at nucleotides 8560–8756 (198 bp) of the *M*. *leonina* mitochondrial genome had significantly higher coverage. This region peaked at a depth of about 8,500 reads and was located in a region containing a long stretch of non-coding nucleotides high in adenine and thymine content and a small portion of the Gln tRNA.

**Fig 2:**
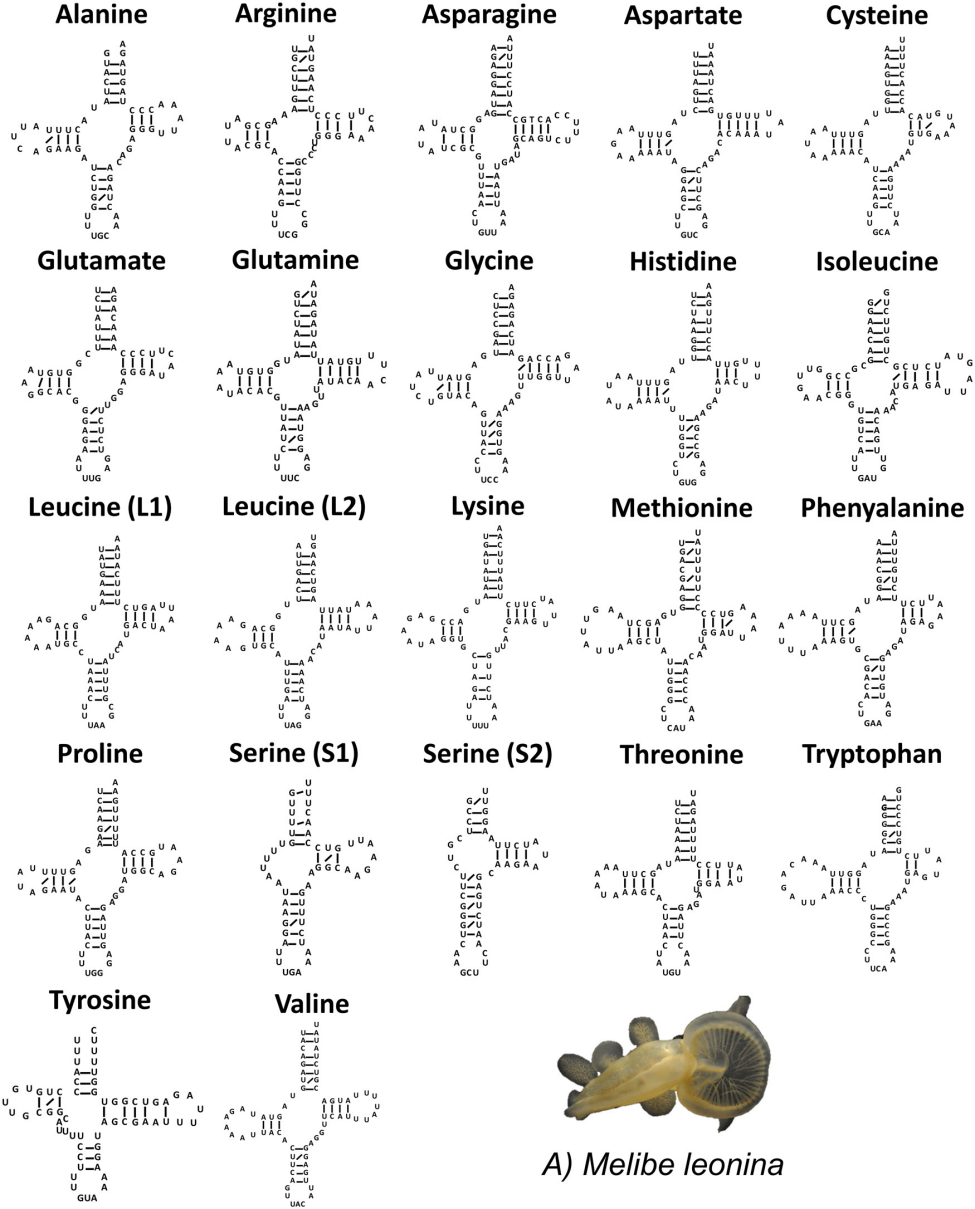
Transfer RNA secondary structures for both *M*. *leonina* (A) and *T*. *diomedea* (B). G-U pair bonds are indicated by a slanted line. The two serine tRNAs had a truncated d arm, seen in other heterobranchs. In *T*. *diomedea* (B), the UCU anticodon for the serine 1 tRNA (highlighted in red) has not been reported in any other gastropod.

Ion Torrent sequencing of the *T*. *diomedea* genome yielded 4,020,000 reads totaling 804Mbp. The mitochondrial assembly from this organism produced a single contig 14,540 bp in length (Fig 1B; KP764765) with coverage of 34X. The sequence was confirmed with dideoxy sequencing and a region of high coverage was identified in the same area as that for *M*. *leonina*. Additional analysis of this region in *T*. *diomedea* suggested that it was actually a complex non-coding repeat region, which likely explains the higher coverage of this region in the assembly for both species. The mitochondrial genomes of *M*. *leonina* and *T*. *diomedea* were 72% identical with only 3872 of the nucleotides variable and 471 gaps between the two. The overall base composition of the mitochondrial genome for both species was also found to favor adenine and thymine. For *M*. *leonina*, A+T content was found to be 64.3% (26.98% A, 37.28% T, 15.07% C, and 20.68% G) and in *T*. *diomedea*, the A+T content was 65.4% (27.80% A, 37.58% T, 14.58% C, and 20.04% G). The *M*. *leonina* and *T*. *diomedea* mitochondrial genomes coded for the expected thirteen protein-coding genes, twenty-two transfer RNAs, and two ribosomal subunits (short and large) that have been seen in related species. The gene order of the thirteen protein-coding genes of *M*. *leonina* and *T*. *diomedea* was identical to that of all other opisthobranchs published in GenBank. When the linear representation of the mitochondrial DNA was split and rearranged, raw reads from the genomic assembly spanned the location of the split and thus confirmed the circularity of both sequences. The location and secondary structure of all twenty-two tRNAs for both species were successfully identified using Arwen v1.2 (Fig 2).

**Fig 3:**
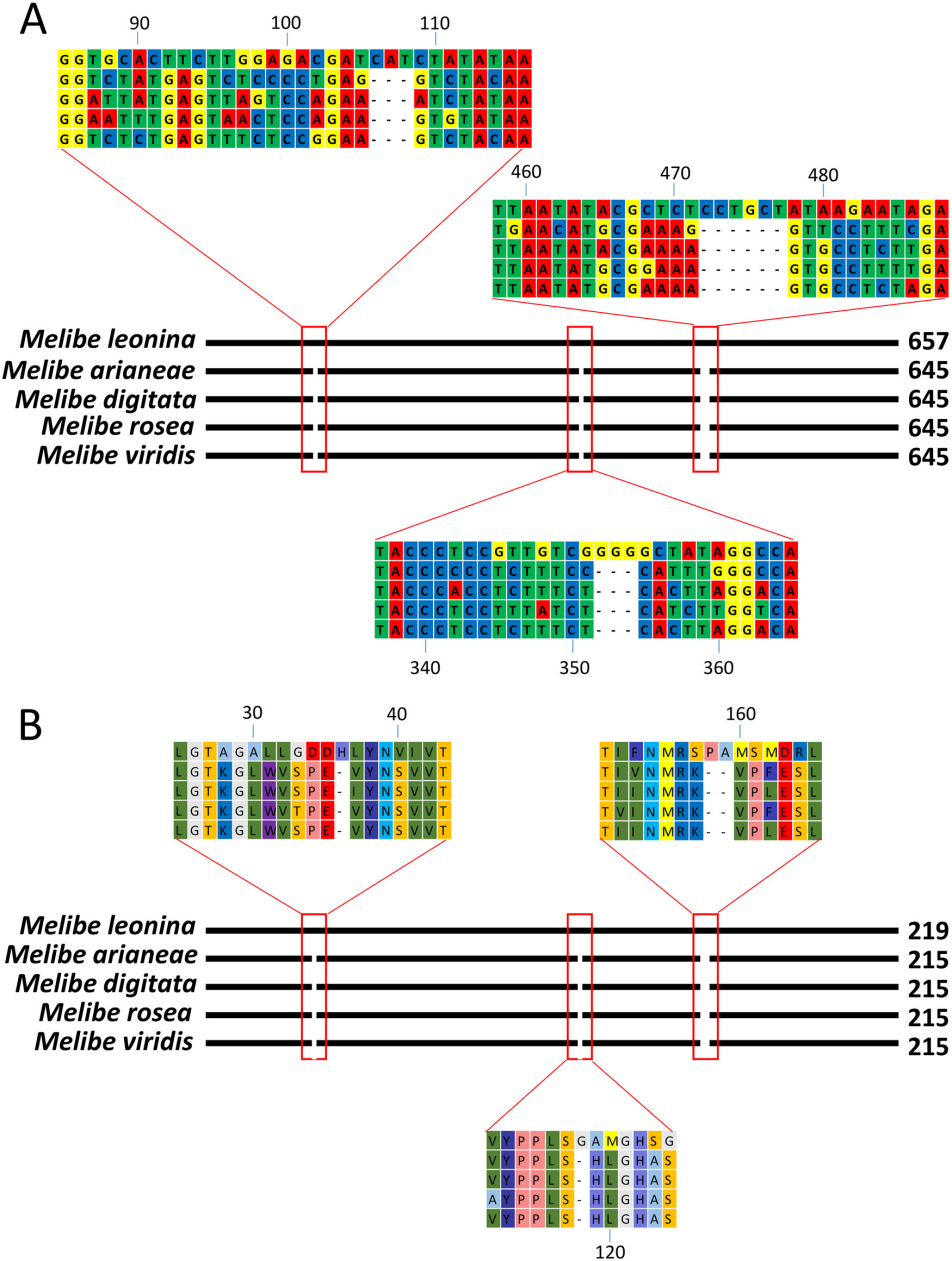
Cytochrome oxidase 1 sequence differences in *Melibe* genus. Nucleotide (A) and amino acid (B) alignments of a portion of the cytochrome oxidase 1 gene for *M*. *leonina* and other members of the *Melibe* genus indicate that *M*. *leonina* lacks the twelve nucleotide deletion present in other species.

The *M*. *leonina* mitochondrial COX1 gene obtained from this study was nearly identical to the *M*. *leonina* COX1 previously available on GenBank (GQ292059). 583 of the 586 nucleotides are identical and the differences did not alter the deduced amino acid sequences. The small ribosomal subunit (16S) was also nearly identical to the previously published 16S sequence in Genbank (GU339202). 436 of the 437 nucleotides were an exact match. When the COX1 sequence of *M*. *leonina* was aligned with all available representatives of the *Melibe* genus in GenBank, *M*. *leonina* did not contain the deletions that were present in the other species (Fig 3). The deletions in the other *Melibe* species were found to span three separate loci; two separate three nucleotide deletions followed by a six nucleotide deletion. A translated amino acid alignment confirmed the locations (Fig 3).

### Phylogenetic analyses

A total of 70 complete gastropod mitochondrial genomes along with a partial mitochondrial genome of *Nerita melanotragus* were available in GenBank and were appropriate for our study. A complete list of gastropod mitochondrial genomes along with current classification, genome length, and GenBank accession numbers can be seen in Table 1. All reported gastropod mitochondrial genomes varied in length between 13,670 and 17,670 bp, with the exception of *Lottia digitalis* which had a mitochondrial genome size of 26,835 bp. Each genome consisted of 22 tRNA sequences, 13 protein-coding genes, and 2 rRNA sequences, with the exception of Lunella aff. cinerea, [8] which contained 23 tRNA sequences. The mitochondrial genome of the patellogastropod *Lottia digitalis* was not included in this study due to a high level of divergence within the mitochondrial genome sequence and a significantly longer genome length than other gastropods.

The resulting alignment of the concatenated nucleotide sequence of the 13 protein-coding genes for the 14 opisthobranchs and Caenogastropoda outgroup (*Lophiotoma cerithiformis*, DQ284754) included 11,450 positions. The nucleotide substitution saturation analyses of the opisthobranch alignment showed that the sequences had little nucleotide substitution saturation and were thus deemed to have adequate phylogenetic signal strength. For the first and second codon positions, the I_ss_ was found to be 0.6075, significantly lower (*p* = 0.0000) than the I_ss.c_ value of 0.8313. The third codon position showed slightly more saturation but still had a significantly lower (*p* = 0.0000) I_ss_ (0.7166) compared to the I_ss.c_ (0.8180). After removal of ambiguity, invariance, and uninformtaive regions using Gblock, 89% of the original 11,450 positions remained.

**Fig 4:**
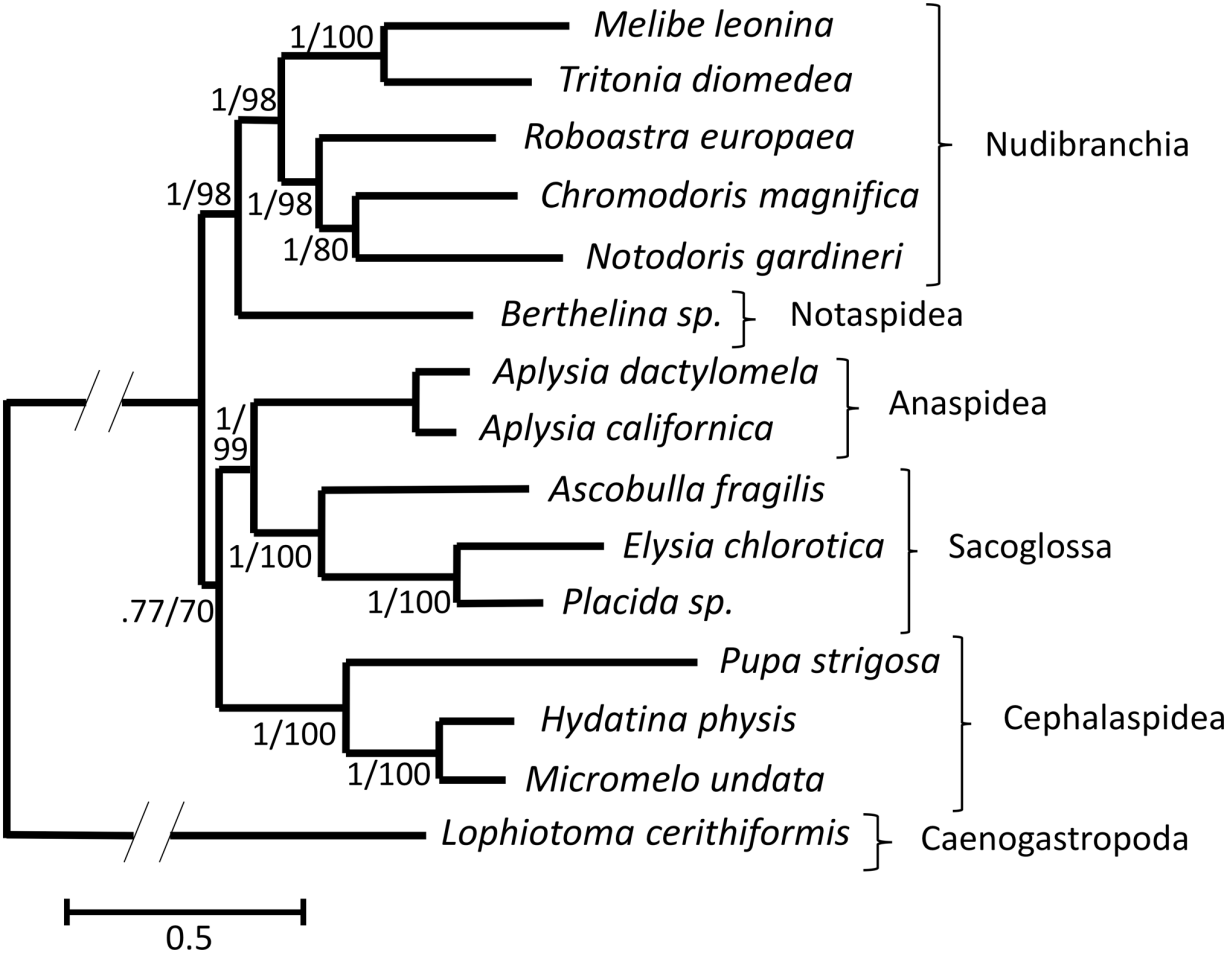
Opisthobranch phylogeny based on nucleotide sequences of the thirteen protein-coding genes of the mitochondrial genome. Both Bayesian and maximum likelihood analyses resulted in identical topologies, shown here as a consensus tree with branch lengths depicted from the maximum likelihood analysis. All sampled orders were found to be monophyletic groups. Numbers at nodes indicate posterior probabilities (Bayesian) followed by bootstrap values (maximum likelihood), indicating statistical confidence in that particular node.

For the opisthobranchs, Bayesian and maximum likelihood analyses resulted in identical topologies with all nodes highly supported and a distinction of five monophyletic orders within Opisthobranchia (Nudibranchia, Notaspidea, Cephalaspidea, Sacoglossa, and Anaspidea) (Fig 4). Nudibranchia was a sister taxon to Notaspidea and together formed a monophyletic group that was sister to the remaining orders. Anaspidea was a sister taxon to the Sacoglossa and together formed a clade sister to the Cephalaspidea. Within Nudibranchia, *M*. *leonina* and *T*. *diomedea* were found to form a monophyletic group separate from the remaining nudibranch taxa, and the previously described Anthobranchian and Cladobranchian clades were supported.

The resulting concatenated amino acid sequence of the 13 protein-coding genes for all gastropods and a bivalve outgroup (*Venustaconcha ellipsiformis*, FJ809753) resulted in an alignment of 4465 positions. *Rapana venosa* had an amino acid identified as “J” which was replaced with an “X” in the alignment. After removal of ambiguity, invariance, and uninformative regions using Gblock, 53% of the original 4465 position remained.

**Fig 5:**
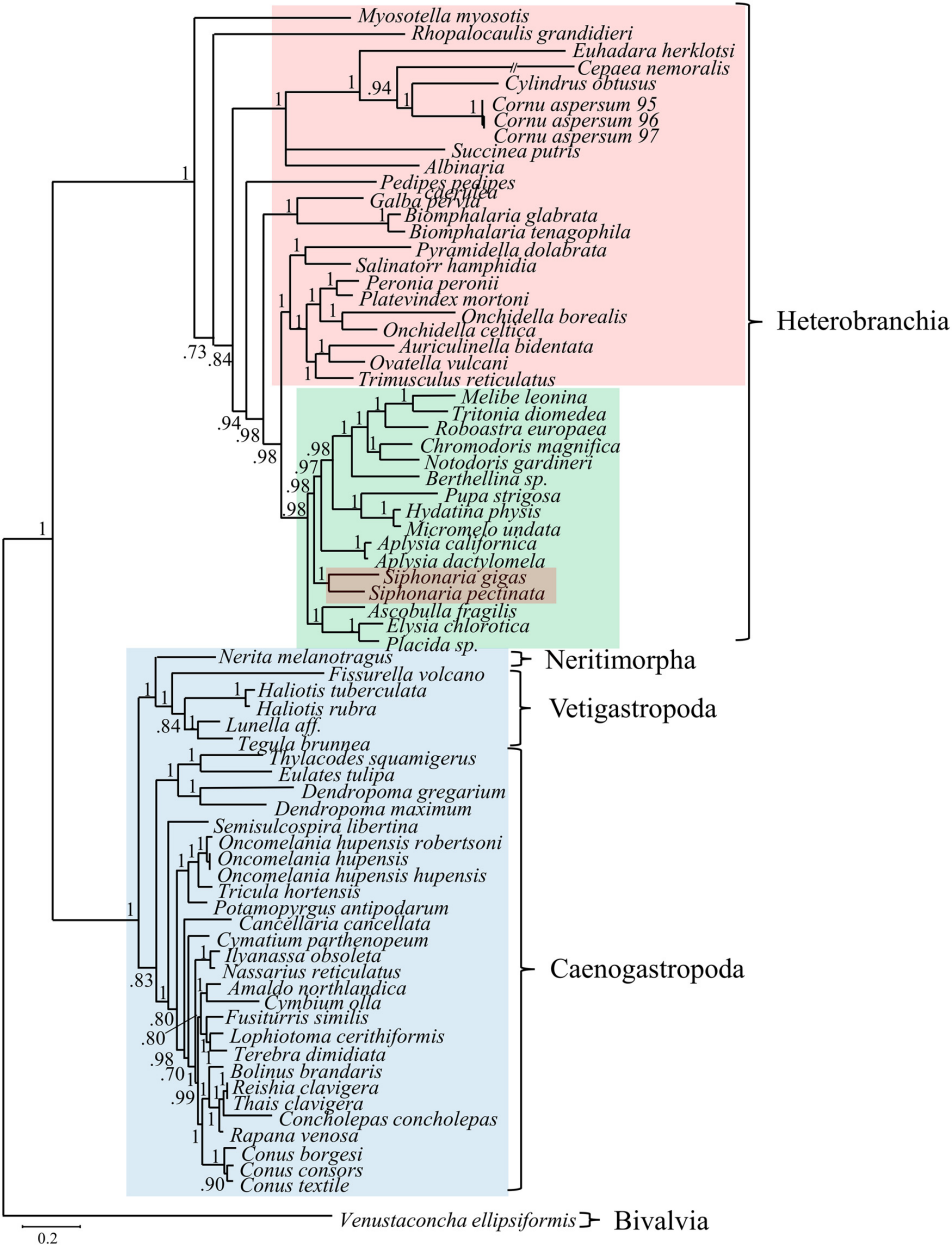
Bayesian analysis of gastropod phylogeny, based on amino acid alignment of 72 gastropods. Posterior probability values indicate the confidence of each node. The bivalve, *Venustaconcha ellipsiformis*, was used an outgroup. The traditional subclasses are highlighted (pulmonates in red, opisthobranchs in green, and prosobranchs in blue). Two of the three traditional subclasses (pulmonates and opisthobranchs) were not monophyletic. In contrast, the four more recently distinguished gastropod groups (Heterobranchia, Caenogastropoda, Vetigastropoda, and Neritimorpha) were all monophyletic.

**Fig 6:**
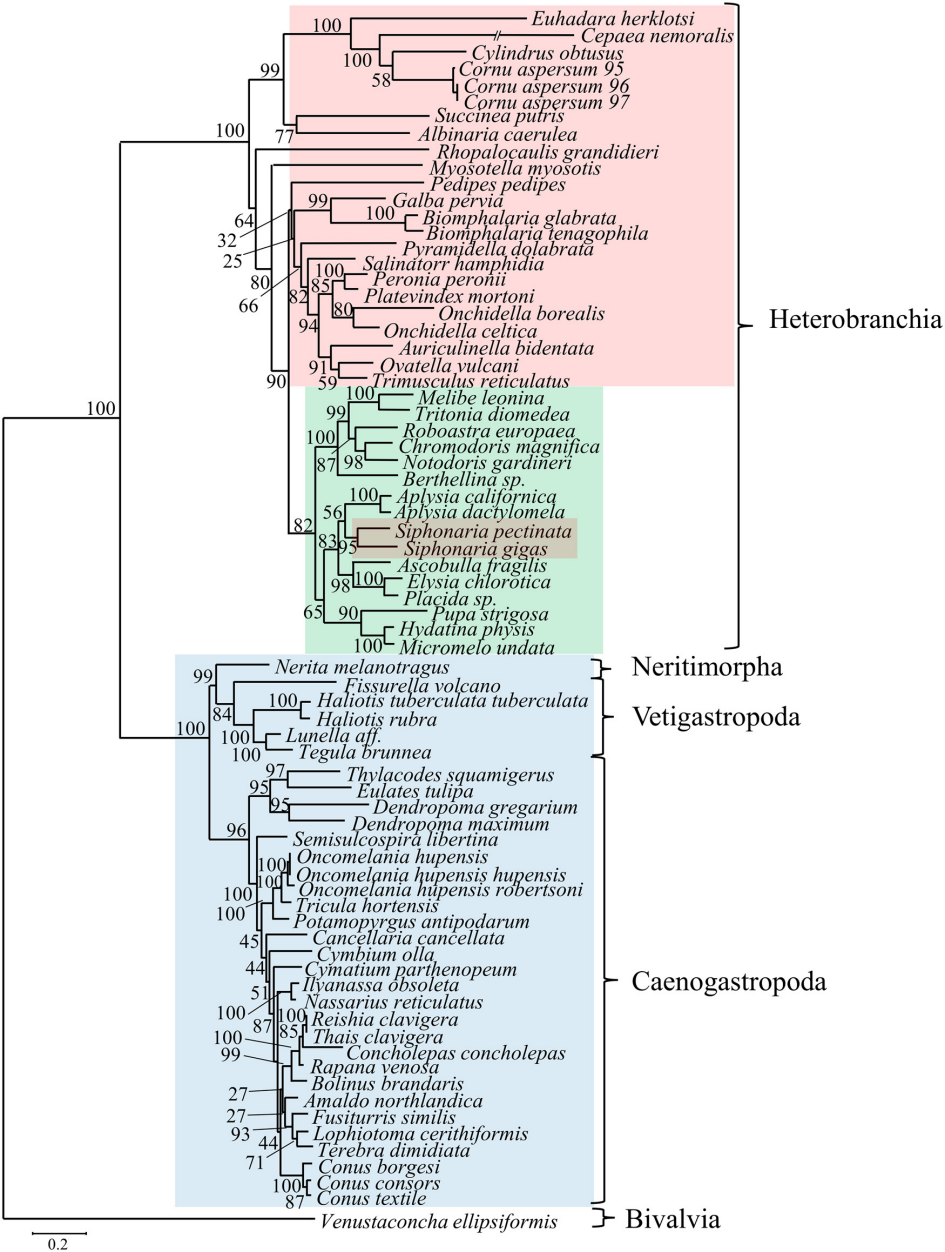
Maximum likelihood analysis of gastropod phylogeny, based on amino acid alignment of 72 gastropods. Bootstrap support values indicate the confidence of each node. The bivalve, *Venustaconcha ellipsiformis*, was used an outgroup. Colors as in Fig 6. While there are minor differences compared to the Bayesian analysis (Fig 5), the topology of major groups is the same (see consensus in Fig 7).

**Fig 7:**
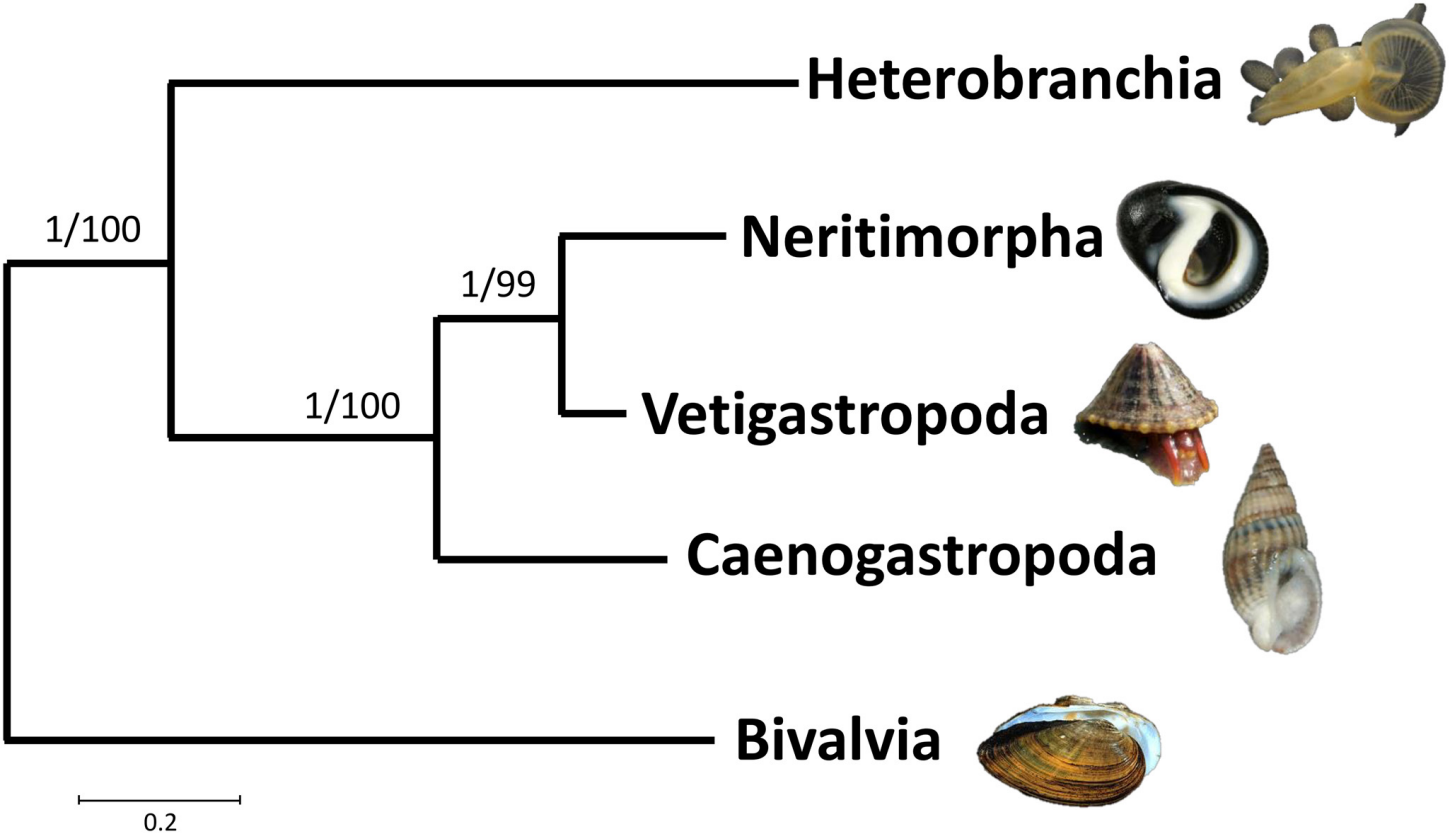
Bayesian and maximum likelihood consensus tree for gastropod phylogeny. All deep nodes for Bayesian and maximum likelihood analyses were identical, and are illustrated here as a consensus tree showing the relationship among the major gastropod groups. The more recently distinguished gastropod groups are all monophyletic and are highly supported. Posterior probability and bootstrap values are located at the nodes.

The Maximum Likelihood and Bayesian analyses of gastropods resulted in nearly identical topologies with all deep nodes highly supported and identical (Figs 5–7). Of the three traditional subclasses, only Prosobranchia was found to be monophyletic because the two pulmonates, *S*. *gigas and S*. *pectinata*, were placed within Opisthobranchia. In contrast, both trees exhibited monophyly of the more recent gastropod divisions: Vetigastropoda, Neritopsina, Caenogastropoda, and Heterobranchia. Heterobranchia, which includes the pulmonates and opisthobranchs, formed a clade that was sister to the other gastropod groups.

## Discussion

### Genome composition and arrangement

Here we present the first two complete mitochondrial genomes from the nudibranch group, Cladobranchia. The two genomes were 72% identical. Like other gastropods (Table 1), *M*. *leonina* and *T*. *diomedea* were found to have relatively small mitochondrial genomes (14,513 and 14,540 bps respectively) compared to many other metazoans, such as *Homo sapiens* (16,569 bp) [35], *Danio rerio* (16,596 bp) [36], *Drosophila melanogaster* (19,517 bp) [37], and another mollusc, *Nautilus macromphalus* (16,258 bp) [38]. Similar to other heterobranchs, these small sizes are likely due to the low number of non-coding regions, the overlap of genes, and the reduced size of genes throughout the genome. The mitochondrial genomes of *M*. *leonina* and *T*. *diomedea* were found to contain the same coding regions, arrangement of genes, and have similar nucleotide content to other nudibranchs [6, 39].

The tRNA structures for *M*. *leonina* and *T*. *diomedea*, were very similar. In both species, the two serine tRNAs contained a truncated d arm. This result is in congruence with the tRNA secondary structures of the nudibranch, *Roboastra europaea* [39], the cephalaspid *Pupa strigosa* [18], and the two pulmonates *Albinaria caerulea* and *Cepaea nemoralis* [40], which also reported to contain the truncated d arm pattern in the two serine tRNAs. These heterobranchs also showed truncation patterns within several other tRNA structures that were not present in *M leonina* and *T*. *diomedea*. In *Lunella aff*. *cinerea*, a representative of the Vetigastropoda, both serine tRNAs have the standard cloverleaf structures [8]. These findings may indicate that the truncated d arm of the serine tRNAs seen in our study is a more derived characteristic that was established in an early heterobranchian ancestor.

In addition, the serine 1 tRNA in *T*. *diomedea* contained an anticodon (UCU) not previously reported in any other gastropod (which have a UGA anticodon). This anticodon is important in distinguishing arginine and serine codons and a mutation within the anticodon may result in a dysfunctional tRNA. Alternatively, *T*. *diomedea* may be an interesting case of an alternative invertebrate mitochondrial genetic code.

The dramatic observation of the deletions seen in the COX1 gene of *M*. *arianeae*, *M*. *digitata*, *M*. *rosea*, and *M*. *viridis* [13, 41] was further clarified in this study, although these deletions were not present in *M*. *leonina*. The shared deletions in these four other *Melibe* species were found to be three separate deletion events, rather than a deletion of twelve contiguous nucleotides, as previously suggested. These deletions are quite surprising because the mitochondrial COX1 protein sequence is highly conserved across species. *M*. *leonina* may be the most basal of all other species within the genus [41, 42] and thus the absence of the COX1 deletion within *M*. *leonina* may indicate that the previously identified deletions are a derived trait and may reflect unusual constraints on the COX1 protein in those taxa. Alternatively, because universal COX1 primers were used to deduce the nucleotide sequence in the other *Melibe* species [13, 41], it is also possible that these universal primers amplified other duplicated copies of the COX1 gene in the mitochondrial or nuclear genomes. In that context, it is noteworthy that the reading frame is not lost in the deleted forms, in spite of the high level of divergence, suggesting the maintenance of protein-coding function. Genome or transcriptome sequencing of these other *Melibe* species might help resolve this possibility.

In the mitochondrial genomes of both *M*. *leonina* and *T*. *diomedea*, the major putative non-coding region had excessively deep read coverage in our assembly. In *T*. *diomedea*, this region appears to be a complex non-coding repeat, which may have resulted in the extensive coverage due to a collapse of repeated sequences during the assembly. If that is the case the number of repeats is estimated to be over 40, based on the depth of coverage in *M*. *leonina*.

### Phylogenetic analyses

In the analyses of opisthobranchs and gastropods, the alignment type (nucleotide vs. amino acid) had a significant impact on the branch support values of the resulting tree. This is similar to other recent analyses of gastropod phylogeny [28]. Opisthobranch nucleotide alignments were found to increase the confidence in the more shallow nodes of the tree, as opposed to the deduced amino acid alignments. When ambiguity was removed with GBlocks, nearly ninety percent of the positions were conserved across the group. In contrast, amino acid alignments were found to be more appropriate for use in the overall gastropod analyses because they increased the confidence in the deep nodes of the group. Roughly fifty percent of amino acid positions were removed with GBlocks prior to analysis of all gastropods. This larger percentage of ambiguity in the gastropod sequences is probably due to the relatively long evolutionary time represented in this group. Regardless, our analyses were based on the most conserved portions of the mitochondrial genomes.

The phylogenetic analyses of opisthobranchs supported the monophyly of five traditional orders Nudibranchia, Notaspidea, Cephalaspidea, Sacoglossa, and Anaspidea. Both Bayesian and maximum likelihood analyses resulted in identical topologies, thus increasing the confidence in the resulting phylogenetic relationships. With the addition of two new Cladobranchian species, the phylogenetic division of Nudibranchia into its two traditional monophyletic clades (Cladobranchia and Anthobranchia) was well supported (Fig 4; 1/98 confidence), which supports many studies using smaller numbers of nuclear or mitochondrial genes [43–47]. Nudibranchia formed a clade with Notaspidea, which was sister to the remaining taxa in the study. This relationship has been reported in many recent studies using nuclear genes [9, 12, 43, 44] and complete mitochondrial genomes [6], and the resulting clade has been named the Nudipleura. The placement of Cephalaspidea, Sacoglossa, and Anaspidea in relation to one another has been contradictory in recent studies examining the groups. Some studies group Anaspidea and Cephalaspidea in a clade together, which is sister to Sacoglossa [6, 43, 45]. Others place Cephalaspidea as sister to the Nudipleura clade with Sacoglossa sister to the remaining groups [28]. Still others have different relationships, although often with a lack of representation of one or more of these orders [7, 8, 9, 44, 46]. In this study, only five of the nine traditional opisthobranch orders were represented. The remaining opisthobranch orders (Acochlidea, Gymonostomata, Rhodopemorpha, and Thecostomata) do not yet have representative species with complete, published mitochondrial genomes. Sequencing the complete mitochondrial genomes of organisms from these remaining taxa and a subsequent phylogenetic analysis will help to address the relationship of these remaining orders within Opisthobranchia. In summary, the results from this study support both the traditional taxonomy of opisthobranchs and more recent findings using complete mitochondrial genomes [10].

Among gastropods, two of the three traditional subclasses, Opisthobranchia and Pulmonata, were not monophyletic and thus appear to be invalid classifications. This finding is due to the placement of the pulmonate *Siphonaria* genus within opisthobranchs, which is identical to other recent findings [10, 28]. *Siphonaria* is a false limpet and has morphological characteristics that differentiate it from opisthobranchs, such as a shell, and thus this finding is in conflict with traditional taxonomy. In contrast, the Euthyneura clade, including both opisthobranchs and pulmonates, is well supported in this study which is in agreement with several recent molecular studies [10, 11, 28, 39]. Additionally, the monophyly of the recently proposed groups within Gastropoda (Heterobranchia, Caenogastropoda, Vetigastropoda, and Neritimorpha) were well supported. This result is in congruence with many morphological and molecular studies [4, 7, 8, 10, 16, 19, 28].

Even though the monophyly of the recently proposed gastropod clades was supported in this study, the particular relationships between these groups does not necessarily agree with some prior studies [14, 48–50]. Of particular note is the fact that many studies support a monophyletic clade that includes the Heterobranchia and Caenogastropoda, referred to as Apogastropoda. Our data do not support this, due to Vetigastropoda and Neritimorpha forming a monophyletic group sister to Caenogastropoda (Fig 7), thus making Apogastropoda paraphyletic in our study. Our data more closely agree with other studies using complete mitochondrial protein-coding sequences of gastropods [8] and mitochondrial gene arrangement [46], grouping Vetigastropoda, Neritimorpha, and Caenogastropoda into a clade that is sister to the Heterobranchia. While the use of complete mitochondrial genomes has been very useful in resolving deep evolutionary relationships in certain taxa [21, 22], some studies suggest that they may be less informative in examining deep molluscan relationships [24]. It may be that our study and that of others using mitochondrial genomes [8, 46] are illustrating the evolution of mitochondrial genomes, rather than indicating accurate evolutionary relationships in these deep nodes. It remains to be seen whether or not this is actually the case, and additional data, such as large-scale genome and transcriptome datasets, may be necessary to resolve these relationships of deeper Gastropod divergences.

Use of complete mitochondrial genomes along with an increased sample size and appropriate phylogenetic model has been shown to increase confidence in phylogenetic inferences of deep evolutionary relationships [8]. Mitochondrial protein-coding genes tend to have a high rate of mutation and are useful tools in addressing recent evolutionary events of closely related species [51]. For the more recently diverged opisthobranch orders, the propriety of mitochondrial protein-coding genes to investigate such relationships was well supported in this study. Bayesian and maximum-likelihood analyses of opisthobranchs resulted in identical, highly supported topologies. In contrast, such a high rate of mutation resulted in only about 50% of the protein-coding genes being highly conserved when all gastropod taxa were aligned together. Even so, deep evolutionary relationships were highly supported and identical between the two analyses. Furthermore, with only 50% gene conservation, the more recent nodes were not as highly supported and identical in both analyses, when examining overall gastropod phylogeny. In order to increase the confidence in these more recent relationships, individual alignments and phylogenetic analyses for each subsequent gastropod group could be used. As seen in the present analyses of opisthobranchs, working with a smaller group removes ambiguity, resulting in better resolution of more recently derived nodes.

Thus far, studies using complete mitochondrial genomes to investigate the taxonomy of gastropods, including the above data, have continued to produce consistent results as the sample sizes have increased. Although this study has more gastropod mitochondrial genomes than any prior study, more taxon sampling is still needed to resolve some remaining taxonomic issues within the class. Sequencing a mitochondrial genome of a representative of Cocculinimorpha has remained elusive and thus this group’s phylogenetic positioning within gastropods is not entirely clear. The lone sample of Patellogastropoda available in GenBank (*Lottia digitalis*, DQ238599.1) had an extremely divergent mitochondrial genome that was significantly longer than any other gastropod. Preliminary analysis with *L*. *digitalis* resulted in this species having a very long branch length and a placement of the Patellogastropoda group sister to Heterobranchia. Furthermore, when *L*. *digitalis* was included in the analyses, the confidence of the remaining nodes fell significantly, due to high divergence of the species from the rest of the dataset. For these reasons, this species was not included in the final analyses reported here. Therefore, we recommend increased sampling of both Patellogastropoda and Cocculinimorpha mitochondrial genomes to further understand the phylogenetic relationships of gastropods.
