# Correction: The Mitochondrial Genomes of the Nudibranch Mollusks, *Melibe leonina* and *Tritonia diomedea*, and Their Impact on Gastropod Phylogeny

**DOI:** 10.1371/journal.pone.0132861

**Published:** 2015-07-13

**Authors:** The PLOS ONE Staff

The publisher apologizes for the following errors that were introduced during the typesetting process.

In the Materials and Methods, under the subheading “DNA isolation, sequencing, and assembly,” the species name is incorrectly spelled as “Melibe leonine.” The correct species name is “Melibe leonina.”

Fig 2B is missing from Fig 2. Please view the complete, correct Fig 2 here.

**Fig 2:**
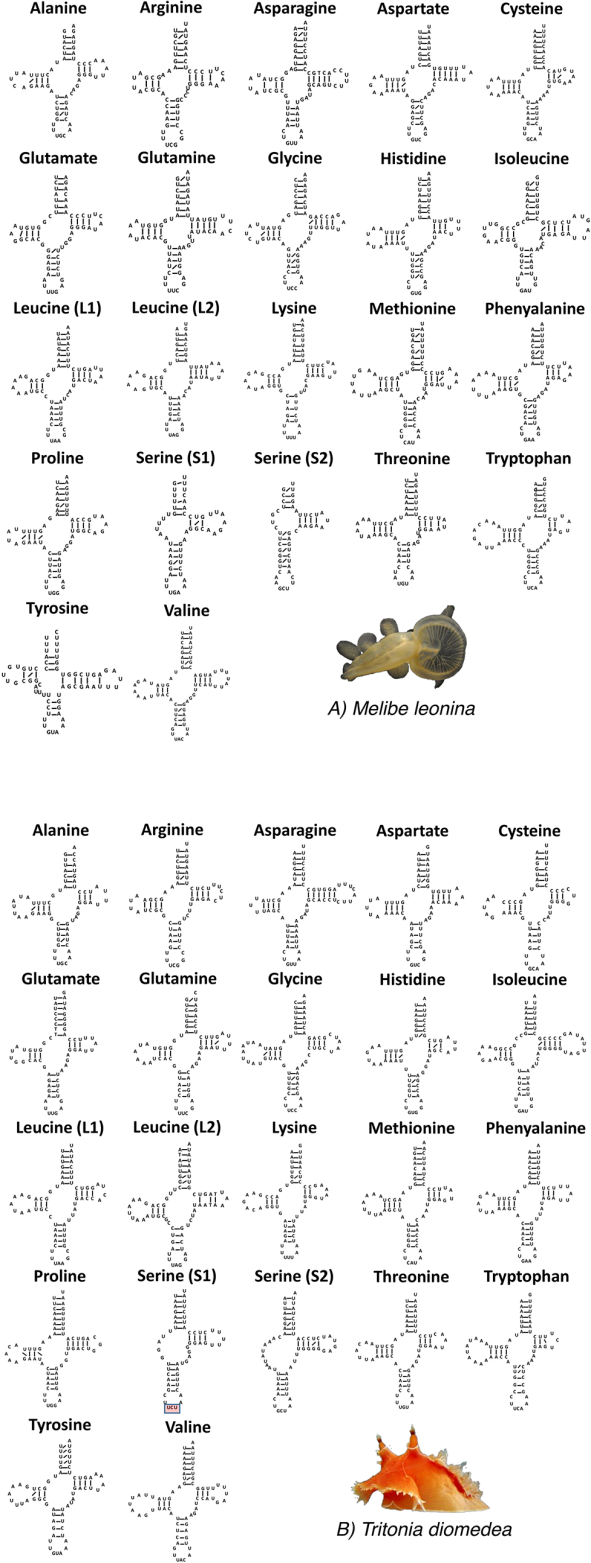
Transfer RNA secondary structures for both *M*. *leonina* (A) and *T*. *diomedea* (B). G-U pair bonds are indicated by a slanted line. The two serine tRNAs had a truncated d arm, seen in other heterobranchs. In *T*. *diomedea* (B), the UCU anticodon for the serine 1 tRNA (highlighted in red) has not been reported in any other gastropod.
